# Cumulative Hepatoprotective, Antihyperlipidemic and Antioxidant Effects of Methanolic Seeds Extract of *Citrullus lanatus* , *Cucumis melo* , *Cucumis sativus* , and *Cucurbita maxima* With GC–MS Profile

**DOI:** 10.1002/fsn3.70358

**Published:** 2025-08-27

**Authors:** Hina Ilyas, Sadia Ghousia Baig, Sana Sarfaraz, Farzana Sadaf, Afshan Siddiq, Ali Asgher, Muhammad Osama, Qudsia Basri, Calvin R Wei

**Affiliations:** ^1^ Department of Pharmacology, Faculty of Pharmacy and Pharmaceutical Sciences University of Karachi Karachi Pakistan; ^2^ Department of Pharmacology, Faculty of Pharmacy Hamdard University Karachi Pakistan; ^3^ Department of Basic Medical Sciences, Faculty of Pharmacy Salim Habib University Karachi Pakistan; ^4^ Department of Pharmacy Practice Faculty of Pharmacy and Pharmaceutical Sciences, University of Karachi Karachi Pakistan; ^5^ Department of Pharmacy Practice Faculty of Pharmacy, Jinnah University for Women Karachi Pakistan; ^6^ Department of Research and Development Shing Huei Group Taipei Taiwan

**Keywords:** antioxidant, fructose, hepatoprotective, high‐fat diet, hypolipidemic, NAFLD

## Abstract

The complex pathophysiological mechanism of nonalcoholic fatty liver (NAFLD) disease has made the prevention and treatment of the disease problematic. The aim of the current study is to evaluate the cumulative hepatoprotective, antihyperlipidemic as well as antioxidant actions of methanolic seed extracts of *Citrullus lanatus* (CL), *Cucumis melo* (CM), *Cucumis sativus* (CS), and *Cucurbita maxima* (CuM) in NAFLD induced by a high‐fat diet along with 20% fructose water in a rat model. A total of 40 male Wistar rats were kept in five groups. Group I (control) rats received 5% Tween 80; Group II (Negative control) rats were given a high‐fat meal with a 20% fructose solution; Groups III and IV received a combination of methanolic seed extract at a dose of 250 and 500 mg/kg, respectively, + high‐fat diet/20% fructose water, while Group V (Positive control) received Atorvastatin (10 mg/kg) and Metformin (200 mg/kg) with a high‐fat diet and 20% fructose. Blood samples were collected at baseline and after 60 days for analysis. The findings demonstrated that, in comparison to Group II, the administration of the combination of seed extracts at the dose of 500 mg/kg substantially (*p* < 0.05) reduced body weight, liver weight, serum cholesterol, TG, LDL, SGOT, SGPT, ALP, and blood glucose. In contrast, HDL, SOD, and CAT levels increased. The results from GC–MS data reveal the occurrence of hexadecanoic acid methyl ester (methyl palmitate) as well as 9,12‐octadecadienoic acid (linoleic acid) in abundance. The presence of these compounds might be responsible for hepatoprotective, antihyperlipidemic, and antioxidant activities.

## Graphical abstract

Assessment of hepatoprotective, antihyperlipidemic, and antioxidant effects of the combination of *Citrullus lanatus* , *Cucumis melo* , *Cucumis sativus* , and *Cucurbita maxima* .

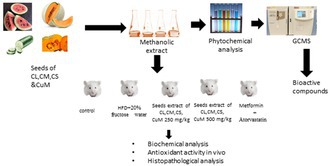

## 1 Background

The nonalcoholic fatty liver represents significant and widespread chronic hepatic disorders globally, constituting a major health challenge in the 21st century (Riazi et al. 2022). This disorder clinically represents high fat deposition in the liver without significant alcohol consumption. It includes a broad category of hepatic conditions that progress from the basic steatosis to hepatocellular carcinoma (Nassir 2022). Its strong link to metabolic syndrome, obesity, diabetes, and insulin resistance has established it as the liver's expression of metabolic dysfunction, reflecting the interaction of genetic, environmental, and lifestyle elements (Neeland et al. 2024). Many researches have proved that diets rich in fats and fructose independently disturb the balance of liver lipids, yet their combined impact is increasingly linked to the onset of NAFLD (Im et al. 2021). An excess of saturated fats surpasses the liver's capacity for β‐oxidation, resulting in the buildup of triglycerides and lipotoxicity. At the same time, fructose, which is primarily processed in the liver, enhances de novo lipogenesis (DNL) by transforming surplus fructose‐derived carbons into acetyl‐CoA, a building block for fatty acid production (Jegatheesan and De Bandt 2017).

The worldwide occurrence of NAFLD has increased substantially, affecting 25% of the population globally, with higher rates in Western nations and among those with metabolic comorbidities (Le et al. 2022). Despite its widespread occurrence, NAFLD remains underdiagnosed and poorly comprehended, often progressing without obvious symptoms until advanced liver damage occurs. This silent progression, combined with the absence of approved drug therapies, emphasizes the critical need for a more comprehensive understanding of the disease's development, risk factors, and potential treatment targets (Mallet et al. 2024).

NAFLD's pathophysiology is complex, involving intricate interactions between lipid metabolism, insulin resistance, inflammation, oxidative stress, and gut microbiome imbalance (Manne et al. 2018). While simple steatosis, which is generally considered harmless, may enhance the chances of developing fibrosis that progresses to nonalcoholic steatohepatitis (NASH), liver cirrhosis, and liver‐related mortality. Despite research advancements, significant gaps persist in our understanding of NAFLD. The mechanisms of disease progression, the influence of genetic and epigenetic factors, and identifying reliable biomarkers for early diagnosis and risk assessment are areas of continuous investigation (Lazure et al. 2022). Furthermore, developing effective prevention and treatments beyond lifestyle changes remains a significant unmet need in the field. In this context, there has been growing awareness about the use of herbal treatments as complementary or alternative therapies for NAFLD. Many traditional medicinal systems have long used plant‐based remedies to treat liver disorders, and recent scientific studies have begun to validate their effectiveness and mechanisms of action (Wang et al. 2023). The justification for exploring herbal drugs in NAFLD lies in their multitargeted mechanisms of action, historical use in traditional medicine, favorable safety profiles, and potential to address the complex pathophysiology of the disease (Wan et al. 2025).

Herbal treatments for NAFLD often target multiple pathways involved in the disease, including lipid metabolism, insulin sensitivity, oxidative damage, and inflammatory conditions of cells (Gong et al. 2024). The plants of the *Cucurbitaceae* family are a remarkable source of biologically active components with diverse pharmacological benefits. The identified components acquire a wide range of biological activities, such as antidiabetic, anti‐inflammatory, anticancer, hepatoprotective, and antimicrobial effects, rendering them extremely helpful for a range of medicinal uses (Mukherjee et al. 2022). This study used the seeds from the Cucurbitaceae family, including *Citrullus lanatus* , locally known as watermelon or Tarbooz; *Cucumis melo* , locally known as melon or kharboja; *Cucumis sativus* (Cucumber); and *Cucurbita maxima* , also known as pumpkin. The reason for the selection of these seeds was based on previously published literature data that has shown the potential biological benefits of these seeds individually. It has been reported that watermelon seeds exhibit significant antioxidant, hypolipidemic, anti‐inflammatory, antibacterial, and hypouricemic effects attributable to the existence of phytochemical constituents in them. The excess amount of oleic acid, along with other phytochemicals in melon seeds, may contribute to their potential health advantages such as anti‐cancer, anti‐inflammatory, hepatoprotective, and immunomodulatory actions. Besides that, studies have also reported their therapeutic potential to treat the conditions of diabetes and heart disease (Zhang et al. 2024). It has been stated in previous studies that *Cucumis sativus* seed extract contains phytosterols that possess hypocholesterolemic properties. It also contains α‐linolenic acid, which is found to have an anti‐inflammatory effect that is indirectly associated with its hepatoprotective properties due to its ability to decrease the formation of ROS and inflammation within the liver (Dimeji et al. 2021). Literature studies have shown that pumpkin seeds contain essential fatty acids, phytosterols, and minerals like magnesium, zinc, and selenium, which help regulate metabolic functions and maintain healthy liver function (Patel et al. 2023). They have antioxidant, antiandrogenic, immunomodulatory, cardiovascular, anti‐inflammatory, and hepatoprotective activity (Wal et al. 2024).

The current study utilized a fatty diet along with fructose solution (20%) in the induction of experimental NAFLD (Inci et al. 2023) and examined their effects on the lipid profile, liver and antioxidant enzymes. While the individual hepatoprotective effects of these seeds have already been investigated against hepatotoxic damage in previous studies (Kumar, Dhanjal, et al. 2024; Lotfi et al. 2023; Mehreen et al. 2024; Onuche et al. 2023), there is a lack of research on their combined use. The current study was planned to explore the cumulative hepatoprotective, antihyperlipidemic, and antioxidative role of *C. lanatus, C. melo, C. sativus*, and Cucurbita maxima.

## 2 Methodology

### 2.1 Seeds Collection

Seeds of *Citrullus lanatus* (CL), *Cucumis melo* (CM), *Cucumis sativus* (CS), and *Cucurbita maxima* (CuM) were purchased from local markets of Karachi and authenticated by the Department of Pharmacognosy, Faculty of Pharmacy and Pharmaceutical Sciences, University of Karachi, with the voucher numbers: CLS‐9‐20, CMS‐10‐20, CSS‐11‐20, and CMS‐12‐20, respectively.

### 2.2 Preparation of Extracts

All the seeds were carefully weighed, cleaned, dried, and roughly ground together before being macerated in analytical‐grade methanol for 21 days. One kilogram of each seed was soaked in three parts of methanol, which were stored in a tightly sealed container and shaken periodically. Following the maceration time, filtration was carried out using Whatman filter paper no. 1 and muslin cloth. At 40°C and lower pressure, extraction was done by utilizing a rotary evaporator. Later, the final extract was preserved and dried in a desiccator to obtain the concentrated extract. The extract was preserved first in a desiccator for 3 days and then transferred to the refrigerator to carry out pharmacological studies. The extract was administered in the form of an emulsion using 5% Tween 80 as an emulsifying agent to ensure dissolution and accurate dosing (Tomer et al. 2024).

### 2.3 Phytochemical Analysis

Each extract was examined by a phytochemical analysis in order to find secondary metabolites, which are involved in a number of biological processes. The qualitative analysis of phytochemicals was done using available established procedures of qualitative analysis, which confirm the presence of various phytochemicals (Ogbuta et al. 2023).

#### 2.3.1 Test for Alkaloids

##### 2.3.1.1 Mayer's Test

2 mL of extract was allowed to react with 2 mL of Mayer's reagent; a yellow cream precipitate formed to ensure that the alkaloids are present.

##### 2.3.1.2 Wagner's Test

Few drops of Wagner's reagent were allowed to react with the 2 ml of test solution. The alkaloids were indicated by the appearance of precipitates of brown to reddish‐brown color.

#### 2.3.2 Test of Phenols

##### 2.3.2.1 Ferric Chloride Test

Small quantity of 5% ferric chloride was added to 10 mg of extract dissolved in distilled water.

Dark green coloration indicated phenols.

#### 2.3.3 Test of Tannins

Two ml distilled water was used to dissolve a small amount of extract, then heated on a water bath. After filtration of this mixture, the filtrate was treated with a few drops of ferric chloride. The presence of tannins was confirmed by the formation of a dark green color.

#### 2.3.4 Test of Saponins

##### 2.3.4.1 Foam Test

0.5 mg of crude extract mixed with 5 mL of water was heated. The appearance of stable foam confirmed that saponins are present.

#### 2.3.5 Test for Flavonoids

##### 2.3.5.1 Lead Acetate Test

10% lead acetate solution (4 mL)was treated with crude extract (2 mL). The presence of flavonoids is confirmed by the formation of a yellow precipitate.

#### 2.3.6 Test for Steroids

##### 2.3.6.1 Liebermann–Burchard Reaction

10 mL of chloroform was added to 200 mg of crude extract. Subsequently, concentrated H_2_SO_4_ as well as acetic anhydride (2 mL) were reacted with this filtrate (2 mL). The formation of a blue‐green ring indicates the presence of steroids.

#### 2.3.7 Test of Terpenoids

##### 2.3.7.1 Salkowski's Test

An amount of 1 g of crude extract was combined with a few drops of chloroform, followed by the gradual addition of concentrated H_2_SO_4_ (2 mL) to form a distinct zone. The terpenoids are confirmed by the emergence of a reddish‐brown coloration.

### 2.4 Determination of Antioxidant Activity by DPPH

With slight modifications, the procedure depicted by (Nahar et al. 2024) was used to evaluate each seed extract's capacity to scavenge DPPH. In short, DPPH reagent (3.5 mL) and 0.5 mL of each extract were mixed together, and the absorbance at 517 nm was measured using ethanol as a blank. The free radical scavenging activity of individual extracts was calculated by formula, and ascorbic acid was used as a reference standard.

$$\text{Radical scavenging}\;\left(\%\right)=\frac{\mathrm{Abs}\;\left(\text{DPPH}\right)–\mathrm{Abs}\left(\text{test}\right)}{\mathrm{Abs}\;\left(\text{DPPH}\right)}\times 100$$

### 2.5 Determination of Phytochemical Compounds in CL, CM, CS, and CuM Seed Extracts by GC–MS

Four seed samples' methanolic extracts were analyzed by using an Agilent Technologies (7890A) GC–MS triple quad system equipped with EI and CI ion sources. The 36‐min GC–MS procedure utilized helium, which is a carrier gas with a column velocity flow set at 1.0 mL/min. The oven temperature started at 60°C and then rose by 10°C each minute, until reaching 310°C, where it remained for 4 min while the injector temperature was kept at 250°C. The GC–MS collection of HEJ was used to compare the retention indices to identify the compound. The mass spectra of the extracts were evaluated against over 62,000 patterns using the National Institute of Standards and Technology (NIST) database (Kalsoom et al. 2024).

### 2.6 Animals

A total of 40 male Wistar rats, weighing between 170 and 200 g, aged 8–10 weeks, were acquired from Dow University of Health and Sciences. These rats were subsequently placed in the animal housing facility of the Department of Pharmacology, University of Karachi. For a week during the acclimatization period, the animals were accommodated in a standard environment (temperature of 22°C ± 2°C, humidity kept in the range of 40%–60%, and 12‐h cycles of light and dark). The rats were freely allowed their diet and water. The HFD was made according to the process explained by Ozkan et al. (Ozkan and Yakan 2019). The University of Karachi's Animal Ethics Committee granted approval for all procedures involving animals (IBC KU‐367/2023).

### 2.7 Chemicals and Drugs

Metformin and Atorvastatin were collected from a reputed pharmaceutical company. L‐ascorbic acid 99%, methanol, and 1,1‐diphenyl‐2‐picrylhydrazyl (DPPH) were acquired from Merck, Germany. Formaldehyde was purchased from the local market. Fructose (GlaxoSmithKline, Pakistan) was used to produce hyperglycemia. SOD and CAT kits were bought from Elabscience Biotechnology Co. Ltd. (Elabscience).

### 2.8 Acute Toxicity

OECD guideline 423 was followed for conducting the acute toxicity research (Patrice et al. 2024).

### 2.9 Study Design

Forty rats were distributed into five groups, with eight rats per group.

Group I (Control) was administered with 5% Tween 80 dissolved in water.

Group II (Negative control) was administered with 40% HFD and 20% fructose solution for 60 days.

Group III Fifteen days pretreated with the combination of methanolic extracts (CL + CM + CS + CuM) 250 mg/kg, followed by HFD/20% fructose water for 60 days (pretreatment followed by induction + treatment of hyperlipidemia).

Group IV Fifteen days pretreated with the combination of methanolic extracts (CL + CM + CS + CuM) 500 mg/kg, followed by HFD/20% fructose water for 60 days (pretreatment followed by induction + treatment of hyperlipidemia).

Group V (Positive control) 15 days pretreated with metformin 200 mg/kg and atorvastatin 10 mg/kg, followed by HFD/20% fructose water for 60 days (pretreatment followed by induction + treatment of hyperlipidemia).

Doses of the extracts were selected on the basis of the results of acute toxicity performed before carrying out this study, while the doses of the drugs were chosen from the previously published studies (Vivekanandarajah et al. 2023). All the dosing was oral for 60 days. Upon completion of the experiment, the rats' body weight was noted down after they had been fasted overnight. The rats were anesthetized by ether and euthanized to obtain blood samples via cardiac puncture.

### 2.10 Estimation of Biochemical and Antioxidant Parameters

The collected blood was placed in sterile, plain centrifuge tubes and allowed to coagulate. Standard methods were followed to assess biochemical parameters. The study evaluated levels of serum glucose, cholesterol, triglyceride, LDL, HDL, SGOT, SGPT, and ALP (Beeton et al. 2007).

A modified standard method was employed to prepare liver homogenate. The entire liver was weighed, then minced and suspended in 15 mL of cold 0.05 M phosphate buffer (pH 7.4) having 0.9% NaCl before being homogenized on ice. The resulting homogenate underwent centrifugation at 10,000 rpm for 10 min, and the supernatant was separated to measure SOD and CAT activities. Standard methods were followed to assess biochemical parameters (Singh et al. 2009).

### 2.11 Histopathology

The pieces of liver were initially extracted and preserved in a 10% formalin solution. Subsequently, it underwent a gradual dehydration process using ethanol concentrations ranging from 50% to 100%. The tissue was then cleared with xylene before being embedded in paraffin wax. Using a rotary microtome (Leica RM 2125 RTS, Singapore), sections measuring 5–6 μm in thickness were cut separately. With the help of dyes, that is, hematoxylin and eosin, these sections were enabled for the microscopic examination of histological changes in the liver tissue (Kumar, Sharma, and Kumar 2024).

### 2.12 Statistical Analysis

The results were represented as mean ± standard deviation. To analyze the average values across multiple groups, a one‐way ANOVA for variance was employed. To compare groups statistically, a one‐way ANOVA followed by Tukey's post hoc analysis test was employed. P‐values < 0.05 were regarded as significant.

## 3 Results

### 3.1 Qualitative Screening of Phytochemicals in the Methanolic Seed Extracts of CL, CM, CS, and CuM

The percentage yields of the seed extracts of CL, CM, CS, and CuM were 22.9%, 23.9%, 22.8%, 22.8%, and 23.5% (w/w), respectively. In accordance with established procedures, the methanolic seed extracts of CL, CM, CS, and CuM were qualitatively screened to identify the presence of active phytochemicals, such as secondary metabolites. The current study's findings for all four extracts revealed a significant class of phytochemicals (Table 1).

**TABLE 1:** Preliminary phytochemical analysis of the methanolic seed extracts of *Citrullus lanatus*, *Cucumis melo*, *Cucumis sativus*, *Cucurbita maxima*.

Constituents	MeOH extract of CL	MeOH extract of CM	MeOH extract of CS	MeOH extract of CuM
Alkaloids	+	+	+	−
Phenols	−	−	−	−
Tannins	+	+	+	+
Saponins	−	−	−	−
Flavonoids	+	−	+	−
Steroids	−	−	−	−
Terpenoids	+	+	+	+

### 3.2 Determination of In Vitro Antioxidant Activity of the Extracts of CL, CM, CS, and CuM by DPPH Assay

**FIGURE 1:**
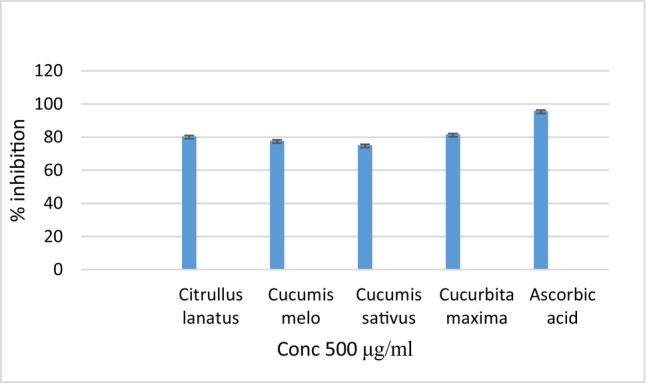
DPPH scavenging activity of the methanolic seed extracts of CL, CM, CS, and CuM.

Antioxidants help in preventing damage to biological systems. These antioxidants can be found in fruits, vegetables, and other wild plants. Using the DPPH test, the antioxidative capacity of natural compounds has been thoroughly investigated. Free radical scavenging effects of the methanolic seed extracts of CL, CM, CS, and CuM are depicted in Figure 1. The antioxidant capability was measured using the DPPH radical inhibition test, and the percentage inhibition of the seed extracts of CL, CM, CS, and CuM was found to be 80.03%, 77.35%, 74.66%, and 81.2%, respectively. Ascorbic acid has the highest antioxidant activity, which is 95.3%.

### 3.3 Determination of Phytochemical Compounds in CL, CM, CS, and CuM Seed Extracts by GC–MS

**FIGURE 2:**
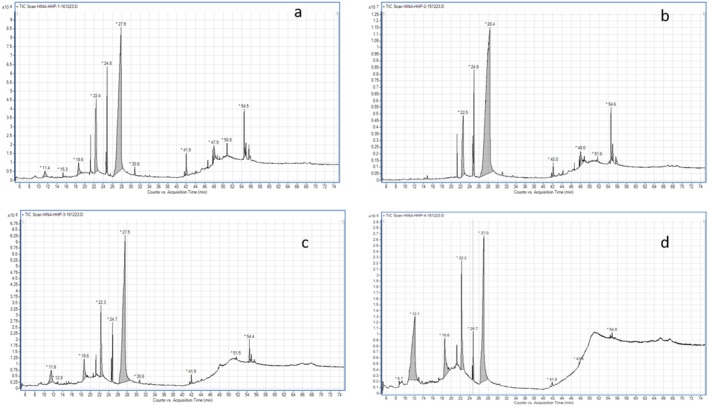
GC–MS chromatograms of the methanolic seed extract of (a) *Citrullus lanatus* and (b) *Cucumis melo* (c) Cucumis sativus (d) Cucurbita maxima.

The gas chromatographic analysis of the individual methanolic seed extract of CL,CM, CuM and CS showed several peaks in the generated chromatograms (Figure 2). The bioactive components in the methanolic seed extracts of CL, CM, CS, and CuM were identified by gas chromatography–mass spectroscopy technique. The names of bioactive compounds, retention time (RT), compound name, molecular weight, and their biological activity are depicted in Tables 2, 3, 4, 5, respectively. GC–MS chromatograms of all seed extracts displayed a dominant peak corresponding to the fatty acid 9,12‐octadecadienoic acid (linoleic acid).

**TABLE 2:** Compounds identified by GC‐MS in the Methanolic seed extract of *Citrullus lanatus*.

RT (min)	Compound name	MF	MW
22.4	Hexadecanoic acid, methyl ester	C_17_H_34_O_2_	270
24.8	9‐Octadecenoic acid	C_21_H_38_O_4_	354
27.9	9‐12 Octadecenoic acid	C_18_H_32_O_2_	280
41.9	Oleic acid,	C_21_H_42_O_2_Si	354
47.9	Dodecanoic acid	C_14_H_28_O_2_	228
50.8	γ‐Tocopherol	C_28_H_48_O_2_	416
54.5	Lupeol	C_30_H_50_O	426
55.5	γ‐Sitosterol	C_29_H_50_O	414

**TABLE 3:** Compounds identified by GC‐MS in the methanolic seed extract of *Cucumis melo*.

RT (min)	Compound name	MF	MW
21.2	Hexadecanoic acid, methyl ester	C_17_H_34_O_2_	270
24.9	9‐Octadecenoic acid	C_21_H_38_O_4_	354
28.4	9,12‐Octadecadienoic acid (Z,Z)	C_18_H_32_O_2_	280
42	Ethyl iso‐allocholate	C_26_H_44_O_5_	436
48	Squalene	C_30_H_50_	410
51.6	1‐Heptatriacotanol	C_37_H_76_O	536
54.6	D:B‐Friedo‐B':A'‐neogammacer‐5‐en‐3‐ol	C_30_H_50_O	426
55.6	Stigmasta‐7,16,25‐trien‐3‐ol, (3β,5α)—	C_29_H_46_O	410

**TABLE 4:** Compounds identified by GC‐MS in the methanolic seed extract of *Cucumis sativus*.

RT (min)	Compound name	MF	MW
8.7	Pyrrolizin‐1,7‐dione‐6‐carboxylic acid, methyl(ester)	C_9_H_11_NO_4_	197
12.1	Oleic Acid	C_18_H_34_O_2_	282
18.6	Hexadecanoic acid, methyl ester	C_17_H_34_O_2_	270
22.2	l‐(+)‐Ascorbic acid 2,6‐dihexadecanoate	C_38_H_68_O_8_	652
24.7	9‐Octadecenoic acid	C_21_H_38_O_4_	354
27	9,12‐Octadecadienoic acid (Z,Z)‐, methyl	C_19_H_34_O_2_	294
41.8	Stigmasterol	C_29_H_48_O	412
47.6	1‐Heptatriacotanol	C_37_H_76_O	536
54.8	Trilinolein	C_57_H_98_O_6_	878

**TABLE 5:** Compounds identified by GC‐MS in the methanolic seed extract of *Cucurbita maxima*.

RT (min)	Compound name	MF	MW
11.6	Falcarinol	C_17_H_24_O	244
18.6	Hexadecanoic acid, methyl ester	C_17_H_34_O_2_	270
22.3	l‐(+)‐Ascorbic acid 2,6‐dihexadecanoate	C_38_H_68_O_8_	652
24.7	Trans‐13‐Octadecenoic acid, methyl ester	C_19_H_36_O_2_	296
27.5	9,12‐Octadecadienoic acid (Z,Z)	C_18_H_32_O_2_	280
30.6	2H‐Benzo[f]oxireno[2,3‐E]benzofuran‐8(9H)‐one, 9‐[[[2‐(dimethylamino)ethyl]amino]methyl]octahydro‐2,5a‐dimethyl	C_19_H_32_N_2_O_3_	336
41.9	Trilinolein	C_57_H_98_O_6_	878
51.5	1‐Heptatriacotanol	C_37_H_76_O	536
54.4	Ethyl iso‐allocholate	C_26_H_44_O_5_	436

The phytochemicals identified in the extract of *Citrullus lanatus* (CL) are hexadecanoic acid methyl ester, 9‐octadecenoic acid, 9–12 octadecenoic acid, Oleic acid, dodecanoic acid, γ‐tocopherol, lupeol, and γ‐sitosterol. The GC–MS result confirmed the presence of hexadecanoic acid methyl ester, 9‐octadecenoic acid, 9,12‐octadecadienoic acid (Z,Z), ethyl iso‐allocholate, squalene, trilinolein, 1‐heptatriacotanol, D:B‐friedo‐B′:A′‐neogammacer‐5‐en‐3‐ol, stigmasta‐7,16,25‐trien‐3‐ol, (3β, 5α)‐ in the extract of *Cucumis melo* (CM). The presence of pyrrolizin‐1,7‐dione‐6‐carboxylic acid, methyl (ester), oleic acid, hexadecanoic acid methyl ester, ascorbic acid 2,6‐dihexadecanoate, 9‐octadecenoic acid, 9,12‐octadecadienoic acid (Z,Z)‐, methyl, stigmasterol, 1‐heptatriacotanol, and trilinolein were also found in the extract of *Cucumis sativus* . Falcarinol, hexadecanoic acid methyl ester, L‐(+)‐ascorbic acid 2,6‐dihexadecanoate, trans‐13‐octadecenoic acid, methyl ester, 9,12‐octadecadienoic acid (Z,Z), 2H‐benzo[f]oxireno[2,3‐E]benzofuran‐8(9H)‐one, 9[[2(dimethylamino)ethyl]amino]methyl‐octahydro‐2,5a‐dimethyl, trilinolein, 1‐heptatriacotanol, and ethyl iso‐allocholate in the extract of *Cucurbita maxima* (CuM) (Tables 2, 3, 4, 5).

### 3.4 Effects on Body and Liver Weight

As displayed in Table 6, at baseline, there were no variations seen in the body weight and the liver weights in all the groups. However, Group II (HFD and fructose water) had a noteworthy gain in body and liver weights in comparison to Group I (control) on the 60th day of the study. The gain in body and liver weights in the treatment groups (III, IV, and V) was notably lower than that of Group II at the end of the study (60th day). Group V (Metformin/Atorvastatin) showed minor body weight gain and liver weight compared to Group IV (500 mg/kg).

**TABLE 6:** Effects of combination of methanolic seeds extract of (CL, CM, CS, CuM) on the body and liver weight.

S. No	Groups	Body weight (g) Day 0	Body weight (g) Day 60	Liver weight (g) Day 0	Liver weight (g) Day 60
1.	Group I	171.12 ± 6.55	225 ± 5.54	4.75 ± 0.33	4.78 ± 0.39
2.	Group II	172.37 ± 4.65	260 ± 5.1*	4.76 ± 0.35	8.23 ± 0.79*
3.	Group III	171.87 ± 5.24	249.5 ± 6.82* ^,^ ^#^	4.82 ± 0.27	6.22 ± 0.622* ^,^ ^#^
4.	Group IV	170.87 ± 7.29	237.25 ± 3.69* ^,^ ^#^	4.73 ± 0.32	5.22 ± 0.62^#^
5.	Group V	171.12 ± 6.55	232.75 ± 4.16* ^,^ ^#^	4.78 ± 0.30	4.97 ± 0.39^#^

* *p* < 0.05 vs. control (group I).

^#^ *p* < 0.05 vs. negative control (group II).

### 3.5 Effects on Blood Glucose

In Table 7, at baseline, all the groups did not exhibit any significant changes in the blood glucose level. After 60 days, the level of blood glucose noticeably increased in Group II when compared to Group I. However, Groups III–V showed relatively less rise in blood glucose level as compared to Group II. The change in the glucose level of Groups IV and V was significantly lower compared to Group III.

**TABLE 7:** Effects of combination of methanolic seeds extract of (CL, CM, CS, CuM) on blood glucose levels.

S. No	Groups	Glucose (mg/dL) Day 0	Glucose (mg/dL) Day 60
1.	Group I	89.87 ± 6.17	89.25 ± 5.62
2.	Group II	90.12 ± 4.15	153.25 ± 8.41*
3.	Group III	89.37 ± 4.77	123.75 ± 5.03^#^
4.	Group IV	89.12 ± 4.15	110.12 ± 4.29^#^
5.	Group V	88.87 ± 5.05	97.25 ± 5.25^#^

* *p* < 0.05 vs. control (group I).

^#^ *p* < 0.05 vs. negative control (group II).

### 3.6 Effects on Lipid Profile

**FIGURE 3:**
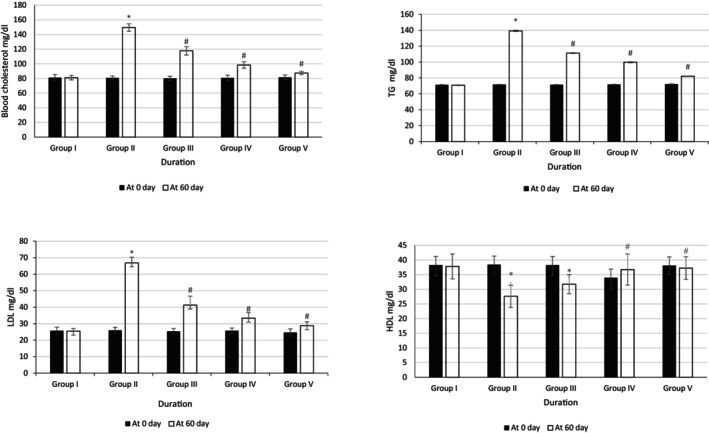
Effects of methanolic seed extract (CL, CM, CS, and CuM) on lipid profile *p < 0.05 vs. control, ^#^ *p* < 0.05 vs. negative control.

Results depicted in Figure 3 show that at baseline there were no changes in the blood cholesterol, triglyceride, LDL, and HDL levels in all the groups when compared with each other. In Group II, the levels of Chol, TG, and LDL significantly increased as compared to Group I after 60 days. The reduction in all the parameters (Chol, TAG, and LDL) of Groups III, IV, and V was also significant as compared to Group II at 60th day of the study.

It was noted that Group II had a significantly decreased level of HDL as compared to Group I. Groups IV and V showed significantly increased HDL levels when compared to Group II. The effect observed in Group V was more significant compared to Group IV.

### 3.7 Effects on Serum SGOT, SGPT, and ALP

**FIGURE 4:**
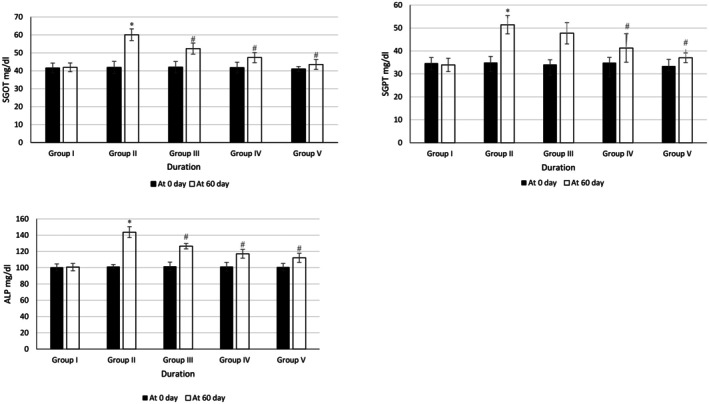
Effects of methanolic seed extract (CL, CM, CS, CuM) on liver enzymes *p < 0.05 vs. control, ^#^ *p* < 0.05 vs. negative control.

In Figure 4, it was noted that the levels of SGOT, SGPT, and ALP in all the groups did not show any noticeable changes at baseline. However, after 60 days, it was seen that Group II had a noteworthy increase in the level of SGOT, SGPT, and ALP as compared to Group I. Groups III–V showed significant reductions in the level of SGOT and ALP as compared to Group II. Group V showed improved SGOT, SGPT, and ALP levels when compared to Groups III and IV.

### 3.8 Effects on Liver Tissue SOD and CAT

In Table 8, there was no significant effect observed in the activity of SOD and CAT in all the groups at baseline. Group II had significantly decreased SOD and CAT levels in comparison to Group I after 60 days. Groups III–V showed significant increases in the levels of SOD and CAT as compared to Group II while there were no significant changes in the levels of CAT when Group V was compared to Group IV after two months of the study.

**TABLE 8:** Effects of combination of methanolic seeds extracts of (CL, CM, CS, CuM) on SOD and CAT.

S. No	Groups	SOD U/mg		CAT U/mg	
		Day 0	Day 60	Day 0	Day 60
1.	Group I	12.06 ± 0.67	12.62 ± 0.38	72.25 ± 4.97	71.37 ± 5.55
2.	Group II	12.56 ± 0.96	4.81 ± 0.11*	71.18 ± 5.95	40.11 ± 2.03*
3.	Group III	13.00 ± 0.65	6.85 ± 0.60*, ^#^	71.31 ± 5.48	49.50 ± 2.20*
4.	Group IV	12.97 ± 0.38	9.97 ± 0.37*, ^#^	70.62 ± 5.35	58.62 ± 3.42*, ^#^
5.	Group V	12.2 ± 1.38	10.96 ± 0.46*, ^#^	70.06 ± 7.12	63.50 ± 4.84*, ^#^

* *p* < 0.05 vs. control (group I).

^#^ *p* < 0.05 vs. negative control (group II).

## 4 Histopathological Studies

**FIGURE 5:**
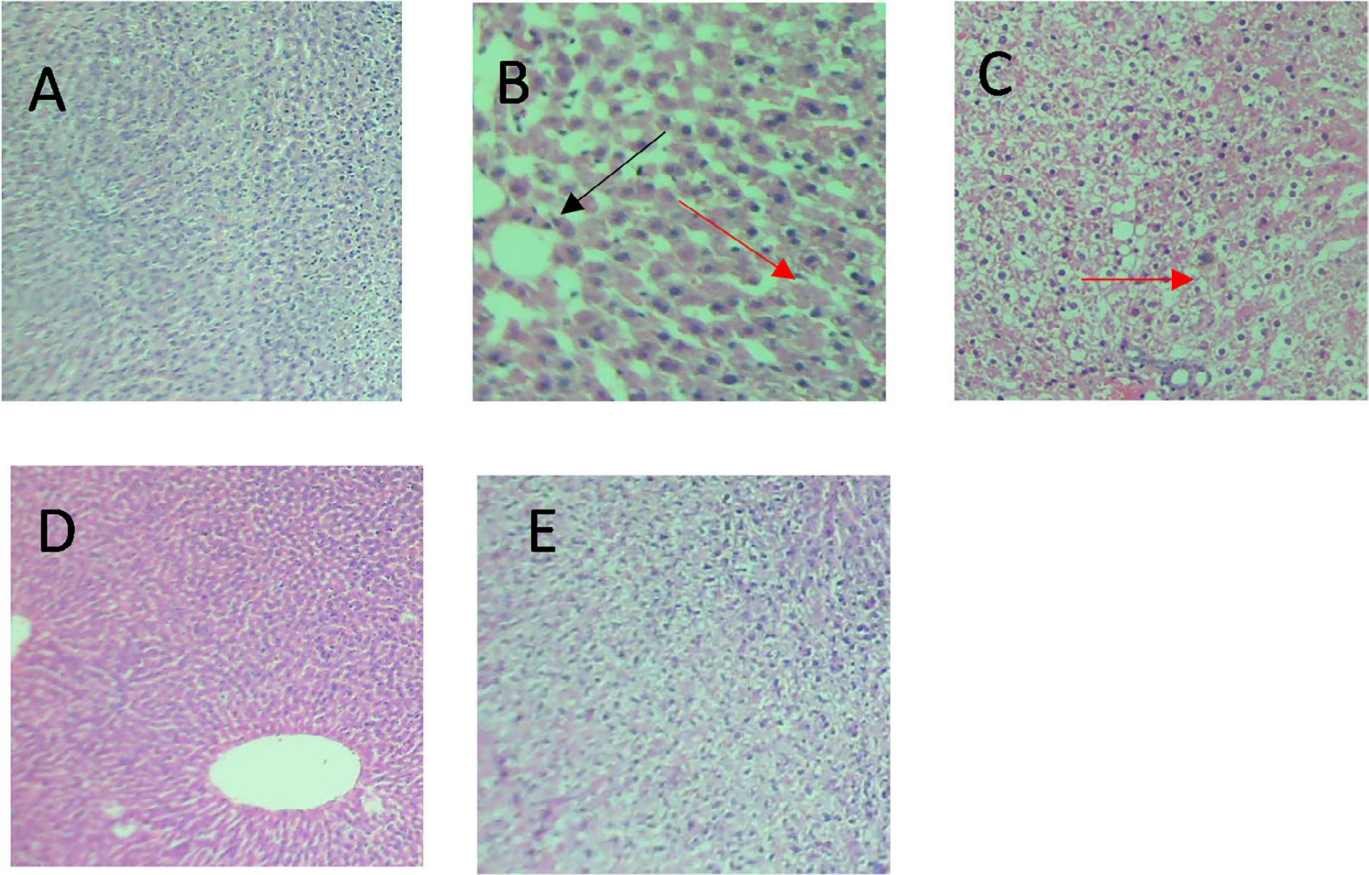
Histopathological changes in the liver of animals induced by HFD and 20% fructose water. Hematoxylin and Eosin (H and E) × 10. (A) In Group I, it shows hepatic parenchyma with an overall intact architecture. No inflammation or steatosis seen. (B) In Group II, which is positive control group, it shows hepatic parenchyma with a focus of ballooning degeneration. (C) In Group III, it showed hepatic parenchyma with an overall intact architecture. Occasional foci of mild inflammation were noted. No steatosis seen. (D) In Group IV, there was hepatic parenchyma with an intact architecture and mild portal inflammation. (E) In Group V, it showed the hepatic parenchyma with an overall intact architecture and mild portal inflammation.

In Figure 5, the liver tissue of Group I shows hepatic parenchyma with an overall intact architecture. No inflammation or steatosis is seen (Figure 5A). In Group II, the high‐fat diet/fructose water group shows hepatic parenchyma with a focus of ballooning degeneration (black arrow) and foci of steatosis (red arrow) (Figure 5B). While Group III shows hepatic parenchyma with an overall intact architecture, occasional foci of ballooning degeneration (red arrow) are noted (Figure 5C). No steatosis is seen. In Group IV, there is hepatic parenchyma with an intact architecture and mild portal inflammation (Figure 5D). In Group V, it shows the hepatic parenchyma with an overall intact architecture and mild portal inflammation (Figure 5E). The intensity of liver tissue damage caused by the high‐fat diet and fructose solution can be seen in Table 9.

**TABLE 9:** Effects of methanolic seed extract combination (CL,CM,CS and CuM) on HFD/fructose‐induced liver damage.

Microscopic features	Group I	Group II	Group III	Group IV	Group V
Fatty change	0	++	0	0	0
Portal inflammation	0	+	+	+	+
Periportal inflammation	0	+	0	+	+
Hepatocyte ballooning	0	++	+	0	0
Hepatocyte necrosis	0	0	0	0	0
Fibrosis	0	0	0	0	0

*Note:* Absent = 0, mild = (+), moderate = (++), severe = (+++).

## 5 Discussion

The high‐fructose, high‐fat (HFHF) diet causes metabolic disorders and increases the chance of developing T2DM, hyperlipidemia, and NAFLD. HFHF causes a significant increase in body weight and hepatic weight (Chen et al. 2024). Similar effects were noted in the present work, when the rats in Group II (HFD/fructose water) significantly gained body weight and liver weight as compared to Group I (control) after 60 days of the experiment. However, the rats of Group V (atorvastatin/metformin) showed a notable reduction in both body and liver weights and exhibited minimal signs of deposition of fat in the liver and in the body. These outcomes are in harmony with the previous research in which metformin is found to lower the levels of LDL and leptin and increase the level of circulating GDF15, which causes appetite suppression, resulting in the reduction of both visceral and overall body fat (Dong and Xu 2024; Dutta et al. 2023; Hamza and Alsolami 2023; Madison 2023). Similarly, the results from Group IV indicated that the combination of seed extracts restricted the increment in liver and body weight. The antiobese effects of the combination of seed extracts might be due to the presence of 9,12‐octadecadienoic acid/linoleic acid in all four seed extracts, as confirmed by the GC–MS in this study. Linoleic acid, by blocking the enzyme HMG‐CoA reductase, has been shown to decrease body weight in human individuals who are predisposed to obesity (Adahıru and Musa 2024; Eke et al. 2021).

A high‐fat diet combined with fructose‐rich water consumption can significantly contribute to hyperglycemia and NAFLD development (Bhat and Mani 2023). In this study, Group II, fed HFD/fructose water, had significantly higher serum levels of glucose compared to Group I (control), while Groups IV and V (metformin/atorvastatin) also showed improved levels of glucose. Literature suggests that Metformin effectively reduces blood glucose levels by primarily inhibiting hepatic glucose synthesis through the activation of AMPK, enhancing muscular glucose uptake, and diminishing intestinal glucose absorption (Augusto et al. 2022; Kim and Lee 2020). Moreover, the Group IV (500 mg/kg) exhibited a lesser rise in serum glucose levels at the end of the experiment, which indicates the presence of antidiabetic and antioxidant compounds in this combination of seed extracts. As it has been proven by different studies that hyperglycemia targets and disturbs several biochemical pathways linked to ROS production (Lee et al. 2024), we can link the glucose‐lowering ability of all the seed extracts to the presence of antioxidant bioactives like Lupeol in the seed extract of *Citrullus lanatus* , which improves insulin sensitivity and controls hyperglycemia by inhibiting the activities of PTP1B, α‐glucosidase, and α‐amylase enzymes (Sen et al. 2024). γ‐Sitosterol, which is detected in the extract of the seeds of *Citrullus lanatus*, significantly reduces blood glucose concentrations by activating the remaining β‐cells, thereby enhancing the release of insulin (Kumari et al. 2023). Hexadecanoic acid, methyl ester, which is detected in all the extracts used in this study, has also been reported to possess anti‐diabetic properties (Mokosuli et al. 2024). It has been proposed that n‐hexadecanoic acid and oleic acid may work together to enhance antidiabetic effects by competitively inhibiting α‐amylase and α‐glucosidase enzymes, thus disrupting the process of carbohydrate conversion into glucose (Agada et al. 2021). There are multiple experimental studies available that validate the anti‐diabetic potential of the extracts of CL, CM, CS, and CuM (Nissar et al. 2025; Vivekanandarajah et al. 2023).

Consuming excessive amounts of fats and carbohydrates, particularly fructose, stimulates hepatic de novo lipogenesis. As a result, the liver experiences an increased accumulation of TGs, contributing to hepatic fat buildup (Régnier et al. 2023). Present study showed that Group II (positive control group) had significantly higher serum levels of cholesterol, triglycerides, and LDL when compared to Group I (control), while Group V (metformin/atorvastatin) exhibited minor changes to the lipid profile after 60 days. These findings provide support for the use of metformin, which significantly lowers visceral and total body fat while also helping obese people with and without diabetes lose weight. By blocking the enzyme that catalyzes cholesterol formation, 3‐hydroxy‐3‐methylglutaryl‐coenzyme A reductase (HMG‐CoA), atorvastatin prevents hyperlipidemia. Previous studies have reported that atorvastatin and metformin effectively decrease TC, LDL, and TG levels while increasing HDL levels in type 2 diabetic dyslipidemias when administered together (Soni et al. 2023). In Group IV (seeds extracts 500 mg/kg), improvements in the lipid profile were observed, which showed the antihyperlipidemic effects of the combination of seeds extract of CL, CM, CS, and CuM. The seeds have demonstrated promising antihyperlipidemic effects in various studies. These seeds contain bioactives such as flavonoids, polyphenols, and unsaturated fatty acids, which contribute to their lipid‐lowering properties. Research has shown that the consumption of these seeds or their extracts can lead to significant reductions in serum cholesterol, LDL, and triglyceride levels. Additionally, they have been found to increase high‐density lipoprotein (HDL) cholesterol levels, further improving the overall lipid profile. The antihyperlipidemic effects of these seeds are attributed to their ability to inhibit cholesterol absorption, enhance lipid metabolism, and reduce lipid peroxidation (Hussain et al. 2022; Aftab et al. 2022; Messaoudi et al. 2019; Shafi et al. 2023; Vivekanandarajah et al. 2023). These reports suggest that incorporating the seeds of *C. lanatus, C. melo, C. sativus*, and *C. maxima* into the diet or developing nutraceuticals from their extracts may offer potential therapeutic benefits in managing hyperlipidemia and associated cardiovascular risks (Dimeji et al. 2021).

Research indicates that consuming diets rich in fat and fructose can trigger oxidative stress, which leads to higher blood levels of SGPT, SGOT, and ALP (Chidambaram and Venkatraman 2010). This stress condition enhances the generation of reactive oxygen species (ROS) while weakening the liver's antioxidant capabilities. The excess ROS causes oxidative harm to hepatic cells, resulting in higher levels of SGPT and SGOT (Banc et al. 2022; Ortega‐Pérez et al. 2024). The current investigation revealed that rats in Group II (HFD/Fructose water) exhibited a notable elevation in SGOT, SGPT, ALP, and bilirubin levels in blood, indicating hepatic injury. However, the groups treated with metformin/atorvastatin showed hepatoprotective effects as they significantly attenuated the liver enzyme abnormalities. Atorvastatin also demonstrated synergistic effects when combined with metformin, showing improvements in liver involvement in metabolic syndrome (Augusto et al. 2022). Literature studies have reported that atorvastatin has been shown to lower the liver enzyme levels, indicating a potential improvement in liver function. These results suggest that atorvastatin may help those who have NAFLD manage their dyslipidemia and liver damage (Eslami et al. 2024). The Group IV (seeds extract 500 mg/kg) showed hepatoprotective effects as they significantly attenuated the liver enzyme abnormalities. These findings are consistent with similar types of studies in which the seeds of watermelon ( *Citrullus lanatus* ) have demonstrated promising hepatoprotective effects in rat models (Erhirhie and Ekene 2013). Pumpkin seed oil is also reported as hepatoprotective in the studies done by Elmeligy et al. in 2019 (Elmeligy et al. 2019). In a recent study, the diet formulated with cucumber was found to reduce the serum liver markers as compared to those investigated in rats fed with a normal diet (Dimeji et al. 2021). Consequently, it can be inferred that the protective effects on the liver observed across all extracts might be linked to their antioxidant‐rich components. Results from the GC–MS data revealed the presence of hexadecanoic acid methyl ester as a common compound detected by the GC–MS in all the extracts. It has been reported for its antioxidant and hepatoprotective activity (Gupta et al. 2023). 9‐Octadecenoic acid is also reported as hepatoprotective and antioxidant (Osman et al. 2024). 1‐Heptatriacotanol possesses antioxidant, anticancer, and anti‐inflammatory effects (Salama et al. 2024).

Studies have shown decreased SOD activity in NAFLD patients, indicating reduced capacity to neutralize superoxide radicals. This decrease may contribute to increased oxidative stress in the liver. Conversely, CAT levels often show a compensatory increase in early NAFLD stages. The imbalance between these antioxidant enzymes plays an important role in NAFLD pathogenesis and progression, highlighting the importance of maintaining redox homeostasis in liver health (Świderska et al. 2019). In the present study, CAT and SOD levels in liver tissues significantly decreased in the HFD/Fructose water‐fed group, indicating excessive oxidative stress. However, pretreatment with the combination of metabolic seed extracts of CL, CM, CS, and CuM at a dose of 500 mg/kg for 60 days in Group IV and with Group V (metformin/atorvastatin) did not significantly disturb CAT and SOD levels as compared with Group 1 (control). The metformin/atorvastatin group exhibited the highest antioxidant effect by improving SOD and CAT levels. This outcome was supported by Augusto et al. 2022, which revealed increased SOD levels and resolution of NAFLD‐related lesions with simultaneous metformin and atorvastatin use. Similarly, the group administered with seed extracts combination (500 mg/kg) boosted SOD and catalase (CAT) activities indicating strong antioxidant potential. Previous literature confirmed this finding, reporting potent individual antioxidant effects of these seeds in various hepatotoxicity models. For instance different extracts of *Citrullus lanatus* seeds have been shown to increase SOD and CAT activity (Bazabang et al. 2023; Messaoudi et al. 2019). *Cucumis melo* seeds' effects on SOD and CAT activities can be inferred from a study where the hydroalcoholic extract of CM seeds demonstrated nephroprotective properties against gentamicin‐induced toxicity in mice, suggesting broader protective effects against oxidative stress‐induced organ damage (Saleem et al. 2019). Similarly, in a study, hydroalcoholic extract of *Cucumis sativus* seeds (HAECS) significantly improved renal function markers and enhanced antioxidant defense (Prasanthi and Adikay 2016). *Cucurbita maxima* seed oil was also found to increase the level of SOD and CAT in a study (Paul et al. 2020). The antioxidant potential of the methanolic seeds extract combination correlates with the presence of different phytochemicals proven by GC–MS, including γ‐tocopherol, lupeol, D:B‐friedo‐B′:A'‐neogammacer‐5‐en‐3‐ol, oleic acid, hexadecanoic acid, hexadecanoic acid methyl ester, squalene, 1‐heptatriacotanol, ascorbic acid, isopropyl linoleate, and stigmasterol.

## 6 Conclusion

This work revealed that a combination of seed extracts from *Citrullus lanatus* , *Cucumis melo* , *Cucumis sativus*, and *Cucurbita maxima* administered at 500 mg/kg exhibited significant lipid‐lowering effects. These benefits were evidenced by decreases in body weight, liver weight, TAG, cholesterol, and LDL, accompanied by enhanced HDL levels. This combination of extracts also exhibited liver‐protective effects, as evidenced by reduced SGOT, SGPT, and ALP levels. Additionally, antioxidant benefits were observed through elevated SOD and CAT levels. The cumulative effects of compounds from these extracts likely contributed to their efficacy. Consequently, the antioxidant properties of *C. lanatus* , *C. melo, C. sativus*, and *C. maxima* extracts show promise in alleviating NAFLD. Future extensive research may lead to the development of a combination‐based nutraceutical with potential for NAFLD prevention.

## Author Contributions

**Hina Ilyas:** conceptualization (equal), formal analysis (equal), investigation (equal), writing – original draft (equal). **Sadia Ghousia Baig:** supervision (equal), visualization (equal). **Sana Sarfaraz:** formal analysis (equal), investigation (equal), writing – review and editing (equal). **Farzana Sadaf:** methodology (equal), validation (equal). **Afshan Siddiq:** resources (equal). **Ali Asgher:** resources (equal). **Muhammad Osama:** resources (equal). **Qudsia Basri:** resources (equal). **Calvin R Wei:** resources.

## Ethics Statement

The University of Karachi's Animal Ethics Committee granted approval for all procedures involving animals (IBC KU‐367/2023).

## Conflicts of Interest

The authors declare no conflicts of interest.

## Data Availability

The datasets generated and evaluated during this study will be available from the corresponding authors on request.
